# Endometritis

**Published:** 1892-12

**Authors:** Clinton Cushing

**Affiliations:** Prof. of Gynecology


### ENDOMETRITIS.

By CLINTON CUSHING, M. D.,
Prof. of Gynecology.

A Clinical lecture delivered at Cooper Medical College, San Francisco, October 5, 1892.

Gentlemen—You have heard the history elicited from these two young women through the questions propounded. They are sisters. The first one has been married five years, has never been pregnant, and has had a leucorrhea since before marriage. She comes to us on account of the vaginal discharge, and because she has a pain in the region of the left ovary.

The other has been married eight years, has had two children and two miscarriages, the last child a year ago, this child having died a month since. She comes to us on account of a leucorrheal discharge and a backache, the discomfort being about the junction of the sacrum and the lumbar vertebræ. She gives a history of not having had leucorrhea before marriage; also, of having nursed a child for eleven months and feeling rather badly and weak as a consequence.

She further complains that sexual relations with her husband cause her a good deal of pain about the uterus. The act is not only painful at the time, but is followed by a sense of exhaustion. She further states, and I would like to call your attention to this point, that she never has had at any time any sexual desire, and still this woman goes on and bears two children, having been four times pregnant, without any sexual appetite. On the contrary, the act has always been disagreeable to her. I speak of this particularly, because I am applied to quite often by married women who are sterile, and tell me they have no sexual desire whatever. They submitted to the act because it was necessary for them as married women to do so. They believed that the absence of the desire had something to do with the fact of their not bearing children. In this case there is, manifestly, no relation between the absence of sexual desire and the aptitude for pregnancy, and this may be considered a fair example which will apply, as a general rule, to these cases.

Now, these two cases are quite common, such as you will be likely to meet with constantly in practice. The first patient tells us that she has had some kind of vaginal discharge since she was a young girl. What, then, was the trouble at the outset? She probably had at the beginning a cervical endometritis, or, in other words, a chronic congestion and thickening of the mucous membrane that lines the neck of the womb. The glands become diseased and enlarged, the secretion markedly increased, resembling the white of an egg as it escapes from the cervical canal, turning to a yellow color in the vagina. This condition is not uncommon even in children, but is much more frequent after puberty.

The second one that we have questioned tells us that she did not have this discharge before marriage, but now the discharge has increased to such a degree as to attract her attention. The first case has pain in the region of the left ovary. A careful examination shows no enlargement of the ovary or of the Fallopian tube, and no displacement of the uterus. It is not always easy to determine the cause of pain in the region of the ovary, where there is no enlargement or displacement of the organ. As you know, at the time of ovulation, the ovary becomes much enlarged, bursts open, an egg escapes, and there is a hemorrhage from the injured surface; in other words, a positive violence is done to the tissue. Should such a process take place on the surface of the body, it would necessarily be attended with very decided pain. That it should produce pain in the ovary in a proportion of cases, seems to me only natural, and it is true that this physiological function which we would naturally expect should be painless, is, in fact, attended in a very large number of women with varying degrees of pain and discomfort. This woman had this pain two years ago. It disappeared and has not returned until now. The sexual relation has nothing to do with the cause of this pain, as she is not living with her husband.

What was the cause of the endometritis in the first case? It is very difficult to say. You ask me what is the cause of the chronic congestion of the mucous membrane that lines the nostrils, in half the children you meet in the street. First comes the question of inheritance. One will give you an account of a drunken father or a dyspeptic mother. Another will tell you about a history of tuberculosis in the family. Another, of having had the measles or scarlet fever when young, and thereafter had chronic congestion of the mucous membranes of the body. Another will tell you that he lived in San Francisco where the wind was cold. Another, that he had indigestion from the beginning, and that was the probable cause. It is so difficult to say in chronic congestion of the mucous membranes what was the original cause. That eruptive diseases, scarlet fever, small-pox and the like, sometimes leave behind them a chronic affection, is well known. That our cold winds here in San Francisco tend to keep up a congestion of the mucous membranes of the body, is also believed. The existence of gout, syphilis, rheumatism, or tuberculosis may all be considered predisposing causes. I think that it is a general opinion among professional men that the younger generation of the people of European origin who live in North America, tend to suffer more from chronic affections of the mucous membranes than their progenitors who remain in Europe. It is said that there is something about the climate of this continent, something about the methods of life, something about the food, something about the general habit, that tends to produce catarrhal affections of the mucous membranes. Now, here is something for you to investigate. Find out why it is so common in this country. It is not confined to San Francisco, Omaha, or New York, but it is generally distributed, all over this country, whether you go to New York, St. Louis, or Philadelphia. If you examine 100 children indiscriminately in the public schools, you will find that a large percentage are suffering from affections of the mucous membranes of the throat and nostrils.

Now, if these causes act upon the mucous membranes of the nostrils and throat, they also act upon the mucous membranes of other parts of the body. These sisters are half Spanish and half Irish —a combination of two very strong races—and both are races that have resisting power. The Irish are a vigorous people, and the Spanish ought to be peculiarly adapted to this climate, because they come from a country almost a facsimile of California. Spain, in all her characteristics, is practically the same as this State, and that is why the Spaniards selected it as their new country. As the English took the country of the north because it was like their own, so the Spanish sought out a country on the new continent similar to their native land, but, as you see, these transplanted and mixed races do not fare much better than others. But what may be some of the local causes independent of the general causes already stated ?

In the first place, we have in women a cause that is in action for about thirty years of their lives. From the time they are fifteen to forty-five, if they are not pregnant or nursing a child, they have a local periodical congestion coming on every four weeks, and attended with a partial exfoliation of the mucous membrane that lines the uterine canal. Now, this repeated congestion of the parts may easily drift into a chronic congestion. It is only necessary that this normal congestion be accentuated by untoward influences in order to produce a chronic endometritis. Let a woman, at the time of menstruation, take unusually long walks, get unusually heated, or expose her legs and feet to unusual cold, the normal congestion of the pelvic organs becomes at once an abnormal one.

What is the argument, then, in this connection ? It is that every woman ought, in justice to herself, to be excluded from every kind of excessive labor, or work, or excitement, at the time of the monthly congestion. I believe that the original man and woman went on all fours. I am convinced that it is through the progress of evolution that we now walk upright on two legs instead of four, and I believe that the woman standing on two legs, with the column of blood having to run up hill from the pelvis, is, to a certain degree, an argument against her power to withstand fatigue that would not exist if she went on all fours. I am quite sure that the woman who stands upon her feet from twelve to sixteen hours out of the twenty-four, and especially during the menstrual period, must suffer, as a consequence, both locally and generally, more than man would suffer under the same hardships. The more delicate the woman, and the poorer the physique, the more marked the evil effect of the upright position for long periods of time. So I say, that the woman cannot make the fight with the world as successfully as the man does. She has this function of ovulation, menstruation, and child-bearing staring her in the face for thirty years of her life, and during which time she ought to be protected and cared for, and be allowed to rest as much as she is disposed.

Constipation is a very common thing with women. If the rectum and the colon are filled with fecal masses, this tends to keep up congestion of the pelvic organs. The pressure on the pelvic veins and upon the ascending vena cava tend to interfere with the return circulation. It is astonishing what an amount of fecal matter a woman will carry around in her abdominal cavity. In preparing women for operations, I find it necessary to give several days to the process of clearing out the intestinal tract ; and, in spite of daily purges, it is found that, after the operation, the woman will sometimes discharge an enormous amount of fecal matter, although in the interim she had taken nothing but fluid food.

It goes without saying, that if you tie a string around your neck moderately tight, you would expect a congestion of the brain. Well, women tie things around their waists, bands, and corsets, and petticoats, which prevent the expansion of the lungs, force the stomach and intestines down into the pelvis, and seriously interfere with the return of the venous blood to the right side of the heart. That the veins of the pelvis, which are poorly supplied with valves, should become dilated and congested, is only natural. Any inflammation of the vaginal mucous membrane, whether simple or specific, may extend into the cervical canal. Any displacement of the uterus backwards or downwards tends to produce congestion and chronic inflammation of the endometrium. Abortions and injury of the cervix during confinement at full term are common causes of endometritis.

What is the prognosis here ? Can these cases of endometritis be cured ? I should say that the chances were fairly good ; that the disorder, under proper treatment, will be greatly modified and probably cured. Much depends upon the habits and environments of the patient. I have found the following plan to give the most satisfactory results : First, I keep the woman under observation for a week, and ascertain by repeated examinations that she is not suffering from tubal disease or chronic pelvic inflammation. She is then placed in bed, an anesthetic administered, a Sims speculum introduced, the cervix seized and steadied with a small vulsellum, the cervical canal dilated with a steel dilator, if necessary, and now with a sharp curette the thickened and diseased membrane is very carefully and thoroughly removed from the entire uterine canal. The cavity of the uterus is then painted over thoroughly with a mixture of equal parts of carbolic acid and compound tincture of iodine. As soon as the effects of the anesthesia have subsided, a bed pan is placed under the woman and a vaginal injection of hot water is administered by the nurse. This injection is repeated every five or six hours until all tenderness and soreness have subsided. The patient is kept in bed for three or four days, after which she is allowed to go about her usual avocations. An application of the carbolic acid and iodine, by means of cotton-covered bamboo sounds, is made twice a week for a month. Treatment is then stopped, and if, at the end of another month, the leucorrhea is not practically cured, the curetting is repeated. Where the disease is slight, the application of the carbolic acid and iodine will often effect a cure after six or eight weeks' treatment. When any application is made to the uterus, I find the glycerine and cotton tampon of decided advantage.

